# Endovascular Treatment of Anterior Circulation Large Vessel Occlusion in the Elderly

**DOI:** 10.3389/fneur.2017.00713

**Published:** 2018-01-19

**Authors:** Mahesh V. Jayaraman, Ryan A. McTaggart

**Affiliations:** ^1^Department of Diagnostic Imaging, Warren Alpert School of Medicine at Brown University, Providence, RI, United States; ^2^Department of Neurology, Warren Alpert School of Medicine at Brown University, Providence, RI, United States; ^3^Department of Neurosurgery, Warren Alpert School of Medicine at Brown University, Providence, RI, United States; ^4^The Norman Prince Neuroscience Institute, Rhode Island Hospital, Providence, RI, United States

**Keywords:** stroke, large vessel occlusion, thrombectomy, elderly, intervention

## Abstract

Endovascular treatment of anterior circulation large vessel occlusion in the elderly population presents special challenges and opportunities. In this review, we discuss the published literature regarding thrombectomy in elderly patients and also discuss specific issues related to treatment in this patient population. In summary, while the overall outcomes following thrombectomy in elderly patients are worse than following thrombectomy in younger patients, there appears to be a similar benefit as in young patients. While there are challenges with successfully delivering thrombectomy in older patients, age alone should not be an independent exclusion from thrombectomy.

## Introduction

Acute ischemic stroke caused by large vessel occlusion (LVO) in the anterior circulation is the leading cause of adult disability in the developed world. Until recently, the only therapy proven to improve outcomes for all ischemic stroke patients was the intravenous (IV) administration of tissue plasminogen activator (tPA), which can be administered up to 4.5 h from the onset of symptoms or when the patient was last known normal. Unfortunately, those strokes caused by occlusion of the large intracranial vessels, such as the internal carotid artery (ICA) and proximal middle cerebral artery (MCA), had low rates of response to IV tPA, and subsequently, poor outcomes (1).

The next revolution in stroke began in 2015, when five randomized trials all showed that rapid mechanical thrombectomy, primarily using stent-retriever devices, significantly improves outcomes in anterior circulation (ICA and MCA) LVO stroke patients (2–7). Even more striking was the absolute magnitude of benefit, with a number needed to treat as low as 2.5 to have one patient be less disabled (8, 9). Few, if any, therapies in medicine can approach that level of benefit. Recently, the benefit of thrombectomy has been shown to extend out to 24 h, in patients with a favorable imaging profile (10).

The application of this highly effective therapy to the older population presents unique challenges, however. The incidence of stroke is higher in the older population (11), and it is likely that we will encounter this issue with increasing frequency in daily clinical practice. In this review, we will highlight these issues in the aging population. We will focus on LVO in the anterior circulation, predominantly in the ICA or MCA.

## Current Literature—Is There a Benefit for the Elderly?

### Single Armed Trials of Treatment

Several single and multicenter case series have been published examining the question of whether there is a benefit of treatment in the elderly (12–16). Sallustio and colleagues compared the outcomes between patients younger than 80 and those 80 and older, in a series of 239 patients. They found no significant differences in clinical outcomes at 90 days with 34.3% of young patients achieving independence (modified Rankin score 0–2) compared with 30.6% of the older group (14). While overall mortality was higher in the older cohort (40.3 vs. 29.2%), the neurologic related mortality was lower in the older group (14.5 vs. 21%), suggesting a larger number of preexisting comorbidities. By contrast, in the multicenter NASA registry, only 27.3% of patients 80 or older achieved independence at 90 days, compared with 45.4% for the younger group (17), suggesting worse outcomes in the older group.

Singer evaluated 362 patients in the multicenter ENDOSTROKE registry and showed a significant dependence of age on outcomes. Only 17% of patients in the oldest quartile (77–94 years old) achieved independence at 90 days, compared with 37% in those 69–67 years old, 47% in the 57- to 68-year-old group, and 60% in those younger than 57. In addition, there was an increasing rate of poor outcome despite recanalization with age, reaching 53% in the oldest age group. They suggested that medical comorbidities, procedural issues such as tortuous anatomy, the use of general anesthesia, and impaired collaterals all played a role in these observed poor outcomes (18). Shi also showed that in a pooled analysis of the MERCI, TREVO, and TREVO 2 trials, increasing age was a strong predictor of poor outcome (19).

Khan and colleagues examined the impact of treatment on nonagenarians, compared with the younger cohort (12). They did show a much lower rate of good outcome in the nonagenarians, with only 11.1% mRs 0–2 at 90 days vs. 48% in the younger cohort, but they acknowledge a higher baseline level of disability in the older group and suggest that there may be a role for therapy in nonagenarians with good prestroke functional status. Recently, Imahori et al. examined the benefit of complete recanalization (TICI 3) vs. incomplete recanalization (TICI 0–2b) in a series of 80 patients, dichotomized by age ≥80 vs. <80 years (15). They found that independence at 90 days (mRs 0–2) was 65% in the older cohort and 68% in the younger cohort, when TICI 3 recanalization was achieved, and 21 vs. 45% for TICI 0–2b in the older and younger cohorts, respectively. This would suggest that perhaps, with complete recanalization, older patients can achieve similar rates of functional independence.

A meta-analysis by Duffis examined this specific issue and found higher rates of symptomatic intracranial hemorrhage and lower rates of good functional outcome in patients older than 80 (20). However, while they included a total of 8 studies with 2,279 patients, there was extreme heterogeneity in the endovascular treatment methods, and most patients were not treated with modern stent-retriever or aspiration devices.

In summary, most single armed trials of thrombectomy in anterior circulation stroke show worse outcomes with older age as compared with younger cohorts.

### Randomized Trials of Thrombectomy

However, all of the aforementioned series were reporting outcomes of patients treated with thrombectomy. What about randomization compared with medical therapy alone? Among the current generation of randomized thrombectomy trials, prespecified analysis based on an age threshold shows significant benefit in older subgroups in the ESCAPE and MR CLEAN trials (divided as 80 years or older vs. younger) and in the SWIFT-PRIME (divided as 70 years or older vs. younger) trial, with no heterogeneity of benefit. In the REVASCAT trial, there was not a difference between medical and endovascular therapy in the older subgroup, but this difference was not statistically significant. These results are summarized in Table 1. The sample size in EXTEND-IA was too small to allow for such an analysis. In HERMES, the pooled, patient level meta-analysis of these five trials, there was no difference in the benefit of thrombectomy across the entire age spectrum using a mixed methods linear regression (8). That is to say, the older patients did worse than the younger patients regardless of treatment modality, but the same degree of benefit of endovascular therapy was present throughout the age spectrum. In addition, when divided into subgroups, the adjusted common odds ratio was not significantly different among the age 50–59, 60–69, 70–79, and 80+ groups, with 218, 333, 371, and 198 patients in those groups, respectively. Similarly, even in the 6–24 h time window, the DAWN trial showed similar benefit to those less than 80 vs. 80 and older, with odds ratios of 1.9 and 2.3, respectively, for benefit with thrombectomy (10).

**Table 1:** Summary of outcomes in older patients in seven randomized trials of modern endovascular stroke thrombectomy (primarily using stent-retriever devices).

Trial	Patients	Odds ratio for favorable outcomes with thrombectomy	Independence at 90 days (mRs 0–2) in older and younger groups; endovascular vs. control
MR CLEAN (6)	500	Age <80 years: 1.6 Age ≥80 years: 3.2	Not reported
ESCAPE (4)	316	Age ≤80 years: 3.0 Age >80 years: 2.7	Age ≤80 years: 59.3 vs. 33.4% Age >80 years: 37 vs. 18%
REVASCAT (3)	206	Age ≤70 years: 2.5 Age >70 years: 0.9	Age ≤70 years: 52.5 vs. 23.3% Age >70 years: 30.9 vs. 34.9%
SWIFT-PRIME (2)	191	Age <70 years: 1.67 Age ≥70 years: 1.78	Age <70 years: 65 vs. 40% Age ≥70 years: 54 vs. 31%
THRACE (7)	402	Age <70 years: 1.6 Age ≥70 years: 1.5	Not reported
DAWN (10)	206	Age <80 years: 1.9 Age ≥80 years: 2.3	Age <80 years: 54 vs. 17% Age ≥80 years: 32 vs. 4%

In summary, while older patients may have worse outcomes overall, there is no heterogeneity of treatment effect seen by age. Thrombectomy is just as effective in elderly patients as it is in younger ones. In addition, the outcomes with medical therapy alone are dismal in older patients, and rapid, successful thrombectomy may by the best chance the older patients have at a favorable outcome.

### Preprocedure: Making the Treatment Decision

Ultimately, deciding to take the patient to the angiography suite for thrombectomy means that the treating team feels the procedure is likely to improve the patient’s chance of meaningful neurologic recovery. In the preprocedure time period, this is dependent on the patient’s baseline functional status, as well as the status of the brain tissue, as assessed by brain imaging.

Almost all the patients in the recent randomized trials of thrombectomy were required to be functionally independent for enrollment, typically defined as modified Rankin scale score of 0 or 1. As such, in patients with preexisting disability, randomized data comparing the outcomes with thrombectomy vs. best medical therapy is lacking. Elderly patients may have a higher rate of preexisting disability, which can make the treatment decision more difficult. Pohjasvaara examined the level of pre- and poststroke disability in a cohort of 486 patients in Helsinki. They found higher levels of prestroke disability in the group aged 71–85 as compared with those aged 55–70 (21). In addition, the older group was more disabled after stroke than the younger group. Another study, compared patients older or younger than 75 and found that indeed the older group had higher prestroke levels of disability, and overall higher mortality after stroke (22). In series of IV thrombolysis in patients with preexisting disability, it has been shown that while the overall mortality is higher, the likelihood of good outcome was not influenced by previous dependency (23). Those authors suggested withholding thrombolysis in patients with preexisting disability might not be justified. Similarly, Leker and colleagues showed that while the overall outcomes following anterior circulation thrombectomy are worse in those with preexisting disability, there were some who were able to maintain their prestroke level of disability, and as such, those with moderate disability should not be excluded from thrombectomy (24). Treatment guidelines from the American Heart Association consider treatment of patients with preexisting disability as “may be reasonable,” but suggest additional data would be helpful (25).

There can be additional difficulties in establishing a functional baseline in patients who come from a non-home living situation. Patients may be in assisted living facilities requiring varying amounts of assistance with activities of daily living. However, in the time critical period between hospital arrival and treatment decision, it may be difficult to elucidate how functional the patient was prestroke, especially when family is not available to provide additional history. These additional items should not delay thrombectomy, but may be difficult to obtain in a timely fashion. It is also possible that patient age may play a role in determining if a patient should be transferred from a non-thrombectomy capable center to one where they can be treated, although data are lacking in this regard.

In summary, preexisting disability is more common in elderly patients and would have excluded them from most randomized trials of thrombectomy. While preexisting disability is not an absolute contraindication to treatment, the team evaluating the patient should do their best to assess prestroke functional status and make an individualized decision in elderly patients with preexisting disability.

### Preprocedure: Imaging Issues

Baseline brain parenchymal imaging (with non-contrast CT) and vessel imaging [with CT angiography (CTA)] should be the minimum standard on all stroke patients, regardless of severity (26). The degree of completed infarction (infarct core) on NCCT is often measured using the Alberta Stroke program early CT score. Core infarct estimation can also be made with assessment of the degree of collateral filling beyond the occlusion on CTA (CTA-Collateral Score), using dynamic CT perfusion, or using diffusion-weighted imaging (DWI) sequences on MRI. With respect to vascular imaging, older patients may have diminished cardiac output, which may result in suboptimal bolus acquisition on CTA. The use of multiphase CTA, with two additional scans through the brain, may ameliorate some of those issues by allowing for additional imaging in those patients with poor cardiac output (27).

Preexisting leukoaraiosis, typically more prevalent in an older patient, has been associated with worse outcomes after thrombectomy as well (28). In addition, leukoaraiosis may lead to overestimation of infarct core in the white matter on CT perfusion using relative cerebral blood flow thresholds (29). Agarwal and colleagues showed in a small series of patients undergoing whole brain CT perfusion that age was negatively correlated with normalized lesion and penumbral volume, but not core volume (30). These changes occurred despite an increased collateral response in older patients and suggest future study is warranted into this age-dependent response to ischemia.

Some series have suggested an age-dependent threshold may be accurate for the degree of baseline infarct beyond which endovascular thrombectomy is unlikely to help the patient (31). A recent series trichotomized patients into age groups, with the oldest group being older than 75 (32). Not surprisingly, the elderly patients had favorable outcomes in 50% of cases with minimal baseline infarction (DWI lesion volume <5 mL), as compared with 80 and 91% of patients with similar lesion volumes in the age 66–75 and age less than 66 cohorts, respectively. More profound, however, was the difference in clinical outcomes when baseline DWI lesion was more than 5 mL, where only 19% of elderly patients achieved independence, compared with 44 and 56%, respectively. This would indeed suggest an age-dependent relationship between baseline infarct and likelihood of a good outcome. The DAWN study also used this concept, with an age-dependent infract core threshold, excluding patients older than 80 if they had a core infarct on CT perfusion or DWI of larger than 21 mL, but allowing infarct cores up to a volume of 50 mL in younger patients.

In summary, when evaluating pretreatment imaging one may need to take patient age into account, especially as it pertains to the evaluation of core infarct volume, regardless of modality.

## Starting the Procedure: Anesthesia and Patient Cooperation

In the HERMES dataset, it was beneficial to use conscious sedation or local anesthesia as opposed to general anesthesia. This effect may be more so in the elderly population, as there are additional risks from anesthesia. Some recent studies, however, have suggested no difference in outcomes between sedation and general anesthesia (33, 34). However, the vast majority of the patients in AnStroke and SIESTA were younger. There can be greater blood pressure decreases in patients under general anesthesia (35), which can be detrimental to collateral flow. Patient cooperation, if the patient is being treated with sedation or local anesthesia, may also be an issue, especially if the patients have some level of preexisting cognitive dysfunction. The use of benzodiazepines, typically diazepam or midazolam is common during procedural sedation. Scholer showed an inverse correlation between age and the dose of both diazepam and midazolam needed to successfully perform endoscopy (36). Interestingly, they showed an even steeper decline in the dose of midazolam needed as compared with diazepam as patients aged.

When using propofol for procedural sedation during endoscopy, Heuss showed that while elderly patients (age >70 years) had slightly higher rates of short periods of oxygen desaturation below 90%, and an overall decrease in oxygen saturation below 5%, especially in those above the age of 85, but they felt that overall, propofol is safe for sedation in the elderly (37).

To summarize, while no study has demonstrated a benefit to using general anesthesia over conscious sedation, nor are there substantial data evaluating the relationship of mode of anesthesia with outcomes in older patients undergoing endovascular stroke thrombectomy, it may be preferable in patients of all ages to use conscious sedation whenever possible.

### Intraprocedure

Intraprocedurally, elderly patients pose anatomic challenges, primarily due to greater tortuosity of their vasculature. Placement of the large bore guiding catheters, including balloon guide catheters, can be more difficult in the elderly population. In extreme cases, direct carotid puncture can be performed, but also likely introduces additional risk. In the NASA registry, procedure times were slightly longer in the elderly cohort, which may be on the basis of the arterial anatomy (17). Imahori also showed longer revascularization times in older patients (45 vs. 31 min) in their single center series (15). In contradistinction, Sallustio showed slightly shorter recanalization times in older patients (60 vs. 78.5 min) (14).

### Postprocedural Care

Post revascularization, critical issues include the need to closely follow the patient’s neurologic exam as well as hemodynamic parameters to potentially minimize the likelihood of intracranial hemorrhage. In elderly patients treated under general anesthesia, postoperative delirium may be common and can cloud the neurologic examination (38).

Patients with higher baseline systolic blood pressure have higher rates of symptomatic intracranial hemorrhage after systemic thrombolysis. As such, current recommendations are to maintain blood pressure below 185 systolic and 110 diastolic following systemic thrombolysis (39, 40). While the optimal target blood pressures after thrombectomy are unknown, a recent retrospective analysis showed that after TICI 2b or greater recanalization, patients in whom the systolic blood pressure was kept below 160 mmHg had better clinical outcomes than those with higher blood pressures (41).

## Future Directions and Unanswered Questions

Among the main unanswered questions, regarding thrombectomy is whether our systems of care are sufficiently well enough organized to provide access to this therapy. Should patients be routed in the field to endovascular capable centers, bypassing closer, non-endovascular capable centers? (42, 43). If so, would there be a bias on the part of EMS to preferentially divert younger patients and not older ones? If such diversion is to occur, is there a differential accuracy of field severity screening tools in younger vs. older patients? One recent study suggests in patients with leukoariosis, severity scores may be less accurate for detection of LVO (44). In addition, a newer trial (NCT02930018) is examining whether a novel neuroprotective agent can improve outcomes in patients undergoing thrombectomy for ICA or M1 occlusions. Will this (or other agents), if shown to be beneficial, have the same effectiveness in older patients?

## Summary

In summary, while the overall benefit of thrombectomy is similar in older and younger patients, elderly patients are less likely to achieve functional independence. This may be on the basis of slightly higher levels of prestroke disability, decreased functional reserve, and diminished tolerance to larger core infarcts before recanalization. Age alone should not exclude patients from thrombectomy, but the treating team should be aware of and prepared for factors, which make treatment in this group potentially more challenging.

## Author Contributions

Both authors contributed to literature review, manuscript revision, and final review.

## Conflict of Interest Statement

The authors declare that the research was conducted in the absence of any commercial or financial relationships that could be construed as a potential conflict of interest.
